# Supplementary material for: Differential distribution of eicosanoids and polyunsaturated fatty acids in the Penaeus monodon male reproductive tract and their effects on total sperm counts
Source: PLoS One. 2022 Sep 22;17(9):e0275134. doi: 10.1371/journal.pone.0275134 (PMC9499254; doi:10.1371/journal.pone.0275134)
Supplement: S1 Table — (DOCX) [file pone.0275134.s001.docx]

**S1** **Table. Percentage of internal standards recovered from liquid-liquid extractions of *P. monodon* testes and vas deferens**

| **Organ** | **Extraction method** | **Percent recovery** | | |
| --- | --- | --- | --- | --- |
|  |  | **PGE_2_-d_4_** | **5(S)-HETE-d_8_** | **EPA-d_5_** |
| Testes | Ethyl acetate | 88.2 ± 4.2 | 85.1 ± 14.7 | 77.1 ± 11.3 |
| Vas deferens | Methanol-chloroform | 80.0 ± 4.3 | 63.5 ± 8.2 | 69.3 ± 8.6 |
|  |  |  |  |  |
|  |  |  |  |  |
