# Supplementary material for: Differential distribution of eicosanoids and polyunsaturated fatty acids in the Penaeus monodon male reproductive tract and their effects on total sperm counts
Source: PLoS One. 2022 Sep 22;17(9):e0275134. doi: 10.1371/journal.pone.0275134 (PMC9499254; doi:10.1371/journal.pone.0275134)
Supplement: S2 Table — (DOCX) [file pone.0275134.s002.docx]

**S2 Table.** **Regression equations for the quantification of PUFAs and eicosanoids in *P. monodon***

| **Compound** | **Linearity range (pg)** | **Linear equation** | **r^2^** | **LOD (pg)** | **LOQ (pg)** |
| --- | --- | --- | --- | --- | --- |
| PGE_2_ | 13.77-1762.50 | y = 0.0004x + 0.0005 | 0.9985 | 16.50 | 50.00 |
| PGF_2α_ | 0.43-110.76 | y = 0.0104x + 0.00005 | 0.9931 | 0.24 | 0.73 |
| 15d-PGJ_2_ | 0.39-197.77 | y = 0.0193x - 0.0011 | 0.9929 | 0.32 | 0.98 |
| (±)8-HETE | 2.93-187.50 | y = 0.0018x - 0.0012 | 0.9857 | 1.83 | 5.56 |
| (±)11-HETE | 2.93-187.50 | y = 0.0033x + 0.0004 | 0.9912 | 0.60 | 1.82 |
| 12(R)-HETE | 1.47-93.75 | y = 0.0063x - 0.0008 | 0.9917 | 0.31 | 0.95 |
| (±)8-HEPE | 2.93-187.50 | y = 0.0020x - 0.0003 | 0.9977 | 1.82 | 5.50 |
| (±)12-HEPE | 1.47-93.75 | y = 0.0062x - 0.000007 | 0.9916 | 0.80 | 2.42 |
| (±)15-HEPE | 1.47-93.75 | y = 0.0056x + 0.0030 | 0.9931 | 1.06 | 3.21 |
| (±)18-HEPE | 1.47-187.50 | y = 0.0034x + 0.001 | 0.9948 | 0.97 | 2.94 |
| ARA | 5.93-380.58 | y = 0.0011x + 0.0002 | 0.9857 | 4.50 | 13.64 |
| DHA | 6.42-821.22 | y = 0.0017x + 0.00081 | 0.9846 | 3.69 | 11.18 |
| EPA | 2.95-756.13 | y = 0.0024x - 0.0009 | 0.9956 | 1.38 | 4.17 |
