# Supplementary material for: Differential distribution of eicosanoids and polyunsaturated fatty acids in the Penaeus monodon male reproductive tract and their effects on total sperm counts
Source: PLoS One. 2022 Sep 22;17(9):e0275134. doi: 10.1371/journal.pone.0275134 (PMC9499254; doi:10.1371/journal.pone.0275134)
Supplement: S3 Table — (DOCX) [file pone.0275134.s003.docx]

**S3 Table. Criteria for the identification of eicosanoids and PUFAs using retention time, precursor ion, proposed fragment ion, and m/z distribution**

| **Compound** | **RT  (min)** | **Precursor ion [M-H]- (m/z)** | **Proposed  fragment ion (m/z)** | **m/z distribution (% relative abundance)** | | | | | | | | | |
| --- | --- | --- | --- | --- | --- | --- | --- | --- | --- | --- | --- | --- | --- |
|  |  |  |  | **1** | **2** | **3** | **4** | **5** | **6** | **7** | **8** | **9** | **10** |
| PGE_2_ | 5.77 | 351.2177 | 271.2067 | 268.9482  (0.3) | 271.2067  (6.4) | 278.5392  (0.2) | 284.4935  (0.2) | 289.2175  (0.2) | 292.4105  (0.2) | 315.1963  (93.2) | 333.2069  (100.0) | 346.8772  (0.3) | 349.3083  (0.2) |
| PGF_2α_ | 5.42 | 353.2333 | 309.2071 | 209.1180  (9.4) | 217.1231  (14.2) | 235.1336  (20.2) | 255.2116  (13.4) | 273.2223  (26.7) | 291.1964  (27.9) | 299.2014  (20.7) | 309.2069  (100.0) | 317.2119  (31.8) | 335.2225  (60.8) |
| 15d-PGJ_2_ | 11.26 | 315.1965 | 271.2067 | 173.3936  (0.5) | 203.1439  (1.2) | 217.1595  (2.3) | 219.1387  (0.3) | 243.1751  (0.3) | 253.1962  (0.4) | 271.2066  (100.0) | 297.1857  (3.1) | 315.1963  (1.3) | 317.2123  (0.3) |
| (±)8-HETE | 12.78 | 319.2278 | 155.0713 | 127.0760  (4.6) | 135.1175  (4.3) | 155.0709  (50.7) | 163.1124  (5.3) | 163.1487  (4.8) | 179.1073  (37.0) | 257.2272  (38.3) | 275.2377  (17.1) | 301.2170  (100.0) | 302.2203  (11.6) |
| (±)11-HETE | 12.46 | 319.2278 | 167.1077 | 167.1073  (100.0) | 167.1437  (4.6) | 168.1107  (6.5) | 195.1023  (6.6) | 211.1337  (5.5) | 257.2273  (6.7) | 275.2378  (27.7) | 291.2328  (13.4) | 301.2170  (40.4) | 302.2204  (5.3) |
| 12(R)-HETE | 12.76 | 319.2278 | 179.1077 | 135.1175  (9.9) | 139.1124  (5.5) | 155.0709  (35.7) | 163.1124  (12.3) | 179.1073  (73.0) | 208.1101  (8.2) | 257.2272  (42.4) | 275.2378  (18.6) | 301.2170  (100.0) | 302.2202  (8.5) |
| (±)8-HEPE | 11.48 | 317.2122 | 155.0713 | 111.0811  (6.7) | 127.0760  (6.6) | 155.0709  (91.2) | 161.1331  (11.2) | 163.1487  (13.1) | 255.2115  (68.2) | 256.2149  (7.3) | 273.2221  (7.9) | 299.2013  (100.0) | 300.2046  (8.8) |
| (±)12-HEPE | 11.51 | 317.2122 | 179.1077 | 135.1175  (13.1) | 137.0965  (4.0) | 163.1125  (5.9) | 163.1485  (3.2) | 179.1075  (100.0) | 207.1025  (10.0) | 208.1105  (33.9) | 255.2115  (30.5) | 273.2225  (16.3) | 299.2015  (41.0) |
| (±)15-HEPE | 11.29 | 317.2122 | 219.1390 | 147.1175  (8.8) | 175.1488  (30.4) | 203.1438  (16.7) | 219.1388  (99.9) | 247.1337  (40.5) | 248.1415  (15.6) | 255.2116  (49.5) | 272.2100  (25.1) | 273.2222  (16.2) | 299.2014  (100.0) |
| (±)18-HEPE | 10.86 | 317.2122 | 259.1703 | 163.1489  (5.9) | 215.1803  (50.1) | 216.1836  (5.7) | 245.1546  (5.7) | 255.2117  (45.6) | 259.1702  (78.7) | 260.1735  (7.9) | 273.2222  (43.0) | 299.2014  (100.0) | 300.2047  (14.1) |
| ARA | 16.69 | 303.2330 | 259.2431 | 173.3933  (2.1) | 205.1959  (17.7) | 210.9558  (0.5) | 231.2117  (0.7) | 259.2430  (100.0) | 260.2464  (2.6) | 285.2223  (5.3) | 301.2170  (0.5) | 303.2327  (53.8) | 304.2361  (18.1) |
| DHA | 16.27 | 327.2330 | 283.2431 | 107.0862  (0.5) | 131.0862  (0.3) | 173.3928  (0.8) | 177.1645  (0.7) | 191.1802  (9.6) | 229.1959  (4.4) | 249.1858  (1.9) | 283.2429  (100.0) | 284.2463  (0.7) | 309.2224  (0.5) |
| EPA | 15.46 | 301.2173 | 257.2275 | 163.1489  (0.5) | 165.1646  (0.8) | 177.1645  (0.7) | 203.1803  (10.8) | 223.1702  (1.6) | 257.2274  (100.0) | 258.2306  (3.1) | 283.2066  (1.8) | 301.2171  (15.4) | 302.2205  (7.1) |
